# Supplementary material for: FgFAD12 Regulates Vegetative Growth, Pathogenicity and Linoleic Acid Biosynthesis in Fusarium graminearum
Source: J Fungi (Basel). 2024 Apr 14;10(4):288. doi: 10.3390/jof10040288 (PMC11051453; doi:10.3390/jof10040288)
Supplement: Supplementary file 1 [file jof-10-00288-s001.zip › Table S1.pdf]

SupplementaryTable

Table S1. The primers used in this study

Primer	sequence (5'–3')
05784-1F	GATGACCGTAGCTGAGAAGC
05784-2R	TTGACCTCCACTAGCTCCAGCCAAGCCAAGGTGGAACGATAATGAAGAG
05784-3F	GAATAGAGTAGATGCCGACCGCGGGTTTAGTCAGTCTCGCTACGGGTTG
05784-4R	AGGCTTGGGCTTAGGTTCAG
05784-5F	TGAGTCTGCTGCCGTTTCT
05784-6R	ACGCTCGTGGTAGTTGTGG
05784-7F	AGCAAGATTCGAGACACAGACTA
05784-8R	AGCAGAATACAGTTTAGTTAGGC
05784hb/F	AGGGAACAAAAGCTGGGTACCTCTCGGTTGAACGCTCTAAT
05784hb/R	GAACAGCTCCTCGCCCTTGCTCACAGCCTTGAGGACGGCAGGAG
pKNTG seq/F	CCCCAGGCTTTACACTTTATGCT
pKNTG seq/R	GTGCTGCTTCATGTGGTCGG
qRT05784-F	GACCCTACCCTTCCCCACTACA
qRT05784-R	CTTGCCCATGACAGGCTTGAT
qRT06184F	CTCAAGCAGAACCCCAAGCGA
qRT06184-R	AGCAGGGAAAGCAAGAGCCAT
qRT07890-F	ATCACCTACCTCCACCACCACC
qRT07890-R	GCCAATGAAGCCAAACTCGC
Tubulin-F	CGTCCAGAGCAAGAACTCATCA
Tubulin-R	TGCGTCGGAACATAGCAGTAA
