# Supplementary material for: FgFAD12 Regulates Vegetative Growth, Pathogenicity and Linoleic Acid Biosynthesis in Fusarium graminearum
Source: J Fungi (Basel). 2024 Apr 14;10(4):288. doi: 10.3390/jof10040288 (PMC11051453; doi:10.3390/jof10040288)
Supplement: Supplementary file 1 [file jof-10-00288-s001.zip › Table S3.pdf]

Table S3. Ergosterol content of PH-1 and *ΔFgfad12* strains

Strain	Content of ergosterol ($\times 10^{-4}$ %)
PH-1	60.98 ± 6.71
ΔFgfad12	87.42 ± 6.37
