# Supplementary figures and images for: FgFAD12 Regulates Vegetative Growth, Pathogenicity and Linoleic Acid Biosynthesis in Fusarium graminearum
Source: J Fungi (Basel). 2024 Apr 14;10(4):288. doi: 10.3390/jof10040288 (PMC11051453; doi:10.3390/jof10040288)

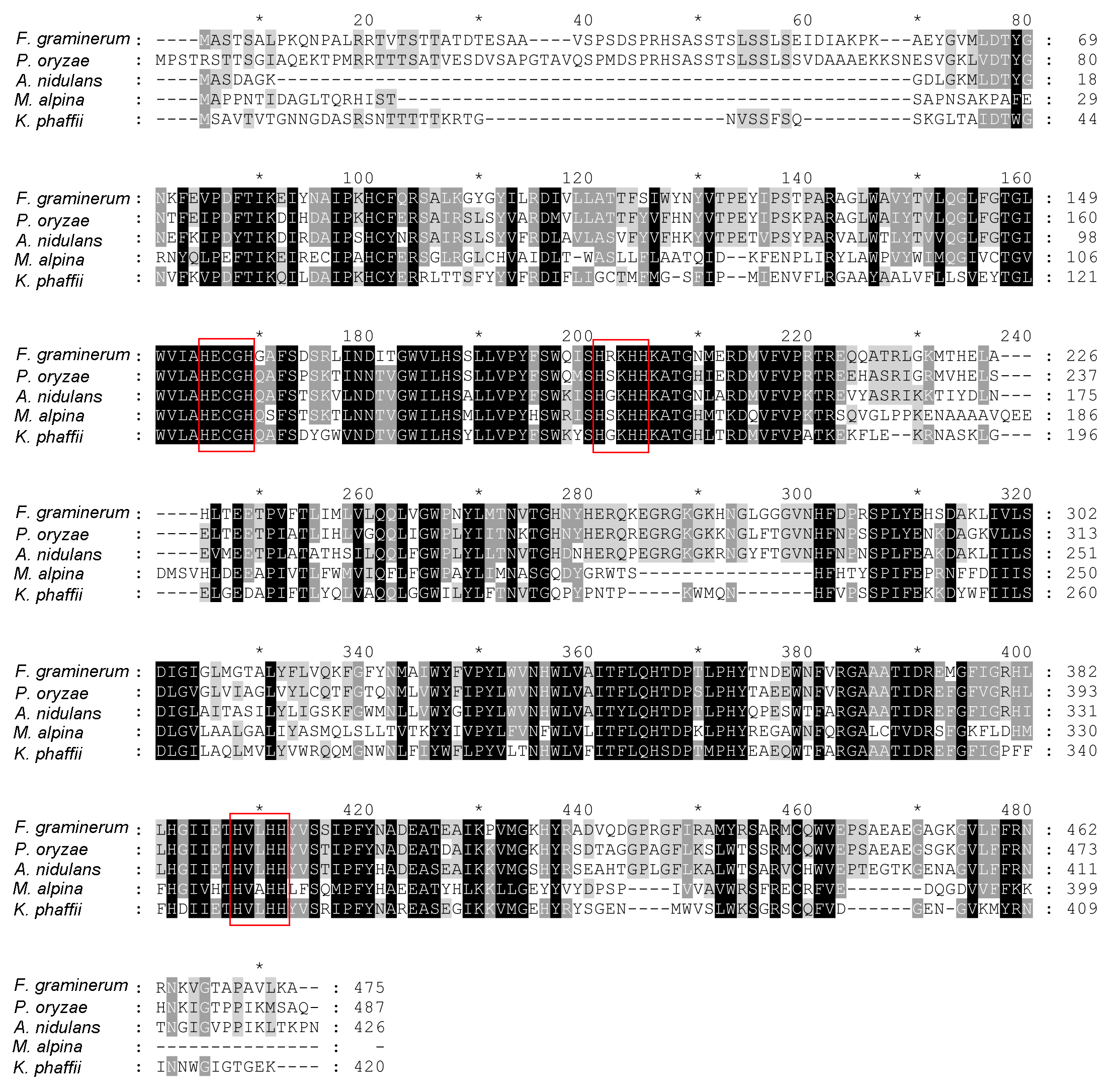

Supplement: Supplementary file 1 [file jof-10-00288-s001.zip › Figure S1.tif]
